# Impact of SARS-CoV-2 pandemic on bariatric care in Poland: results of national survey

**DOI:** 10.1186/s12893-020-00990-7

**Published:** 2020-12-03

**Authors:** Piotr Major, Tomasz Stefura, Michał Wysocki, Piotr Małczak, Anna Rzepa, Monika Proczko-Stepaniak, Jacek Szeliga, Michał Pędziwiatr

**Affiliations:** 1 2nd Department of General Surgery, Jagiellonian University Medical College, Jakubowskiego 2 st., 30-688 Krakow, Poland; 2 Department of General, Endocrine and Transplant Surgery, Medical University of Gdansk, Gdansk, Poland; 3 Department of General, Gastroenterological, and Oncological Surgery, Collegium Medicum Nicolaus Copernicus University, Torun, Poland

**Keywords:** Bariatric surgery, COVID-19, SARS-CoV-2, Pandemic

## Abstract

**Background:**

The SARS-CoV-2 pandemic has reached Poland on March 4th, 2020 and undoubtedly affected all areas of life and medical care, including bariatric care. The study was planned to identify the impact of the SARS-CoV-2 pandemic on bariatric care in Poland.

**Methods:**

The online survey was designed and distributed to bariatric surgeons. The questionnaire was divided into three parts: demographic characteristics of participants and their bariatric centers, examining the impact of the pandemic on the bariatric care and last part with questions about planned care after the pandemic.

**Results:**

49 surgeons participated in the survey. 27 (55%) participants worked in hospitals transformed into COVID-dedicated units. Only 9 (18%) respondents declared uninterrupted bariatric surgery during a pandemic. 91% of surgeons declared continuation of bariatric care with telemedicine techniques. All participants declared a high willingness to resume bariatric surgery after the SARS-CoV-2 pandemic and responded that bariatric procedures should resume immediately when World Health Organisation (WHO) announces the end of a pandemic regardless of oncological treatment. 90% of respondents believe that the pandemic will not affect the safety of bariatric procedures in the future.

**Conclusions:**

Access to bariatric care during the pandemic is limited and redirected to telemedicine. Surgeons are ready to resume bariatric operations immediately after the pandemic, but its end is difficult to determine. In surgeons’ opinion pandemic will not affect the safety of bariatric surgery in the future. The extended waiting list and financial aspects will be the main issues after the pandemic.

## Background

The demand for bariatric surgery in Poland is growing annually, despite a constant increase in the number of conducted surgeries. Currently, in Poland more than 30 surgical centers perform bariatric procedures, adding up to around 5000 procedures throughout the country annually [1].

In December 2019, China became the center of an outbreak of pneumonia caused by a new pathogen named Severe Acute Respiratory Syndrome Coronavirus 2 (SARS-CoV-2) [2]. On March 11th, 2020 World Health Organization (WHO) declared coronavirus disease (COVID-19) global pandemic [3]. The virus has also reached Poland on March 4th, 2020, causing over 9,000 infections and over 350 deaths (Polish Ministry of Health, April 19th, 2020).

Obesity plays an important role in the development of a severe course of SARS-COV-2 infection and is considered as a very strong single risk factor for death [4]. International Federation for the Surgery of Obesity and Metabolic Disorders (IFSO) proposed recommendations for metabolic and bariatric surgery during the COVID-19 pandemic to minimizes risk for the patients and healthcare teams [5].

Currently, data on the impact of the pandemic on bariatric care is scarce. Furthermore, there is no information or any ideas about how to deal with bariatric patients after the SARS-CoV-2 pandemic dissipates.

Our objective was to identify the impact of the SARS-CoV-2 pandemic on bariatric care in Poland.

## Methods

### Study design

An anonymous, online, voluntary open-survey was designed and distributed by the mailing list to all bariatric surgeons associated with the Metabolic and Bariatric Chapter at the Association of Polish Surgeons (SCMiB). An invitation to the project was also published on the official website of SCMiB on April 8th, 2020. E-mail and invitations both included a link to the online form including the survey. The questionnaire was designed by the member of the science committee of the SCIMB. Technical functionality of the electronical questionnaire was tested prior to the study by bariatric surgeons working in first authors department. The survey was conducted between 8 and 15 April 8th and 15th, 2020. Comprehensive instructions on how to complete the survey were included at the beginning of the questionnaire and in the invitation to the project. The questionnaire included multiple choice and Likert scale questions. The order of questions was randomized in each part of the survey. Participants were not offered incentives to participate in the study. Adaptive questioning was not found to be necessary in this questionnaire. Each page included one question, therefore questionnaire included 44 pages. It was not possible to submit an incomplete survey, each question included a “Not applicable” answer. Respondents at any point were able to change their answers by using a “Back button”. Only completed questionnaires were analyzed. Our internet form service did not allow for recording unique site visitor, view rate and participation rate. Authors conducted a log-file analysis to prevent multiple entries from the same individual. The study was designed and described regarding all CHERRIES checklist points for reporting results from internet e-surveys [6].

### Inclusion and exclusion criteria

The study group included surgeons and general surgery residents who are members of SCMiB working in bariatric centers. Both certified bariatric surgeons and non-certified were included in the study. We excluded retired surgeons, surgeons not performing bariatric procedures, and medical doctors who do not practice medicine during the pandemic (sick leave, vacation).

### Survey

The questionnaire was divided into three parts: demographic characteristics of participants and their bariatric centers, examining the impact of the pandemic on the bariatric care and last part with questions about planned care after the pandemic.

- First part gathered data on age, sex, stage of surgical training—resident/specialist/ certificated bariatric surgeon, type of hospital—academic/state hospital/municipal hospital, numbers of bariatric procedures performed in 2019, number of bariatric surgeries performed in 2020 before the outbreak of SARS-CoV-2, actual status of the hospital during pandemic—COVID dedicated hospital/COVID non-dedicated hospital, and information if COVID-19 patients were treated in the hospital.
- Questions in the second part concerned the impact of the pandemic on bariatric care. They asked about the level of current bariatric care, still performed bariatric procedures, numbers of postponed surgeries, reasons for cancellation, the ability to provide the emergency procedure in bariatric patients. Survey included questions about telemedicine and possible application in bariatric care during pandemic, acceptance of this form of patient-doctor communication, and satisfaction with this kind of medium. Roles of surgeon, dietitian, psychologist, and patient organizations online support during pandemic were evaluated with the numeric linear scale (1–10).
- The third part of the survey requested information on bariatric care after pandemic: impact of the pandemic on patients’ waiting list, plans on how to resolve the problem with a large number of suspended surgeries, willingness to resume bariatric surgery after pandemic (scale 1–10). Next questions regarded when bariatric procedures should be resumed, and how to reconcile them with oncological procedures waiting list. At the end of the survey, there were questions about the impact of SARS-CoV-2 on future qualification rules, type of performed bariatric surgery proposed to the patients and their safety after the pandemic.

The questionnaire was developed for this study and it is presented in Additional file 1.

### Ethical considerations

The designed survey was fully anonymous. Personal data of participants collected during study, was not disclosed at any stage. Participants were informed about the length of time of the survey, range of data, that were stored and where and for how long, the name of the primal investigator and the purpose of the study. The study was performed in accordance with the ethical standards laid down in the 1964 Declaration of Helsinki and its later amendments (Fortaleza). Participants were informed about the aim of the study and informed consent was obtained electronically prior to the beginning of the survey. The study was approved by the Bioethics Committee of the Jagiellonian University (1072.6120.103.2020).

### Statistical analysis

Results are presented as numbers with percentages, means with standard deviation or medians with interquartile range, when appropriate.

## Results

### Participants

Overall, 49 surgeons and surgical residents participated in our survey, which constitutes 60% of a total number of SCMiB members (82). Completion rate was 100%. The study group included 41 (84%) males and 8 (16%) females. The mean age was 43 ± 10 years. The group of respondents included 19 (39%) surgeons with bariatric certificate, 19 (39%) surgeons and 11 (22%) residents (Table 1).

**Table 1:** Basic characteristics

	All
n (%)	49 (100%)
Mean age, years ± SD	43 ± 10
Males/females, n (%)	41/8 (84%/16%)
*Stage of surgical training*	
Specialist surgeons with SCMiB certificate, n (%)	19 (39%)
Specialist surgeons, n (%)	19 (39%)
Residents, n (%)	11 (22%)
*Type of hospital*	
University hospital, n (%)	22 (45%)
Voivodship specialist hospital, n (%)	9 (18%)
District hospital/city hospital, n (%)	18 (37%)
*COVID status*	
COVID-dedicated hospital	27 (55%)
Non-COVID dedicated hospital	22 (45%)
*Numbers of bariatric procedures*	
Median number of bariatric procedures in 2019 (January, February), n (IQR)	27 (22–50)
Median number of bariatric procedures in 2020 until pandemic (January, February), n (IQR)	30 (24–50)

### Hospitals

Most of the participants worked in academic hospitals—22 (45%), 18 (37%) worked in district/city hospitals and 9 (18%) in state departments. 27 (55%) of participants worked in hospitals transformed into COVID-dedicated units. 28 (57%) participants declared that patients with SARS-CoV-2 were treated in their hospitals. Median number of bariatric procedures in 2019 (January, February) was 27 (22–50). Median number of bariatric procedures in 2020 until pandemic (January, February) was 30 (24–50) (Table 1).

### Current status of bariatric care

**Fig. 1:**
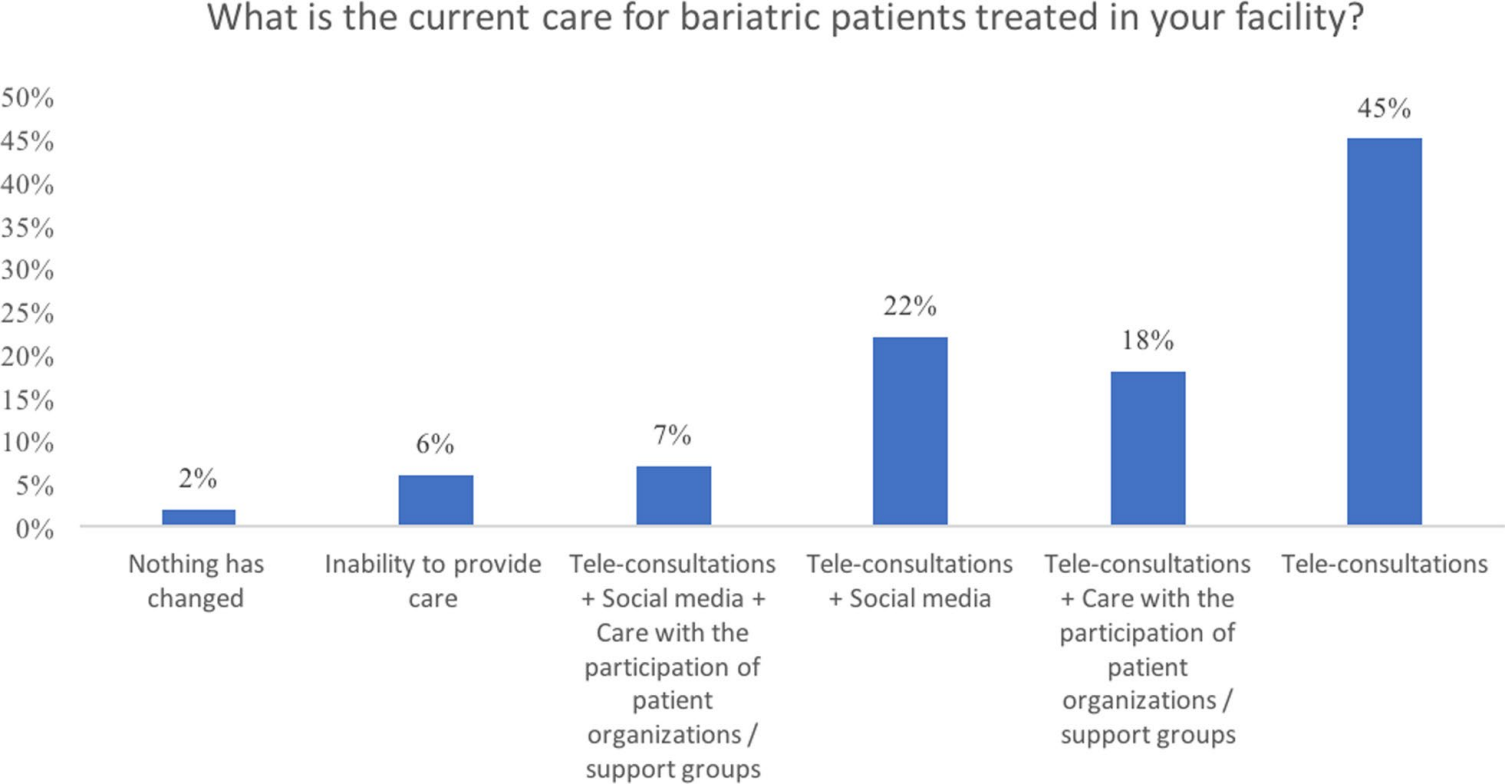
The current care for bariatric patients

Only 9 (19%) respondents declared uninterrupted bariatric service during pandemic, all of whom worked in non-COVID dedicated hospitals. In 100% of COVID-dedicated hospitals, bariatric surgeries have been suspended. Median number of postponed procedures per centre was 23 (18–32). In 40 (81%) respondents the cessation of these surgical procedures was dictated by the hospital management, 8 (16%) by internal arrangements in the surgery department, and 1 (2%) by independent decision of the surgeon. All respondents declare that the limitation of bariatric surgery during pandemic is necessary, median score was 10 (8–10). In the part dedicated to the current care for bariatric patients, 3 (6%) respondents declared inability to provide any care, 1 (2%) respondent declared that nothing has changed, 45 (92%) declared individual phone contact, e-mail, teleconsultations, and social media as an actual way of caring for bariatric patients (Fig. 1). 11 (23%) participants performed full outpatient bariatric service, 38 (77%) performed only follow-up after surgery without qualification of new patients. All respondents consider it necessary to continue providing remote care for bariatric patients. The majority, 45 (93%) respondents, believe that patients have limited access to diagnostic tests ordered by the attending surgeon required before the surgery and during follow-up. During any type of contact with patients, 37 (75%) surgeons informed patients about obesity being a risk factor for a severe course of COVID-19 and death. Only 18 (37%) participants recommend changes in diet, physical activity, and psychological support during pandemic. 43 (87%) surgeons declare that hospital is ready in case of emergency in bariatric patients.

### Bariatric care after pandemic

**Fig. 2:**
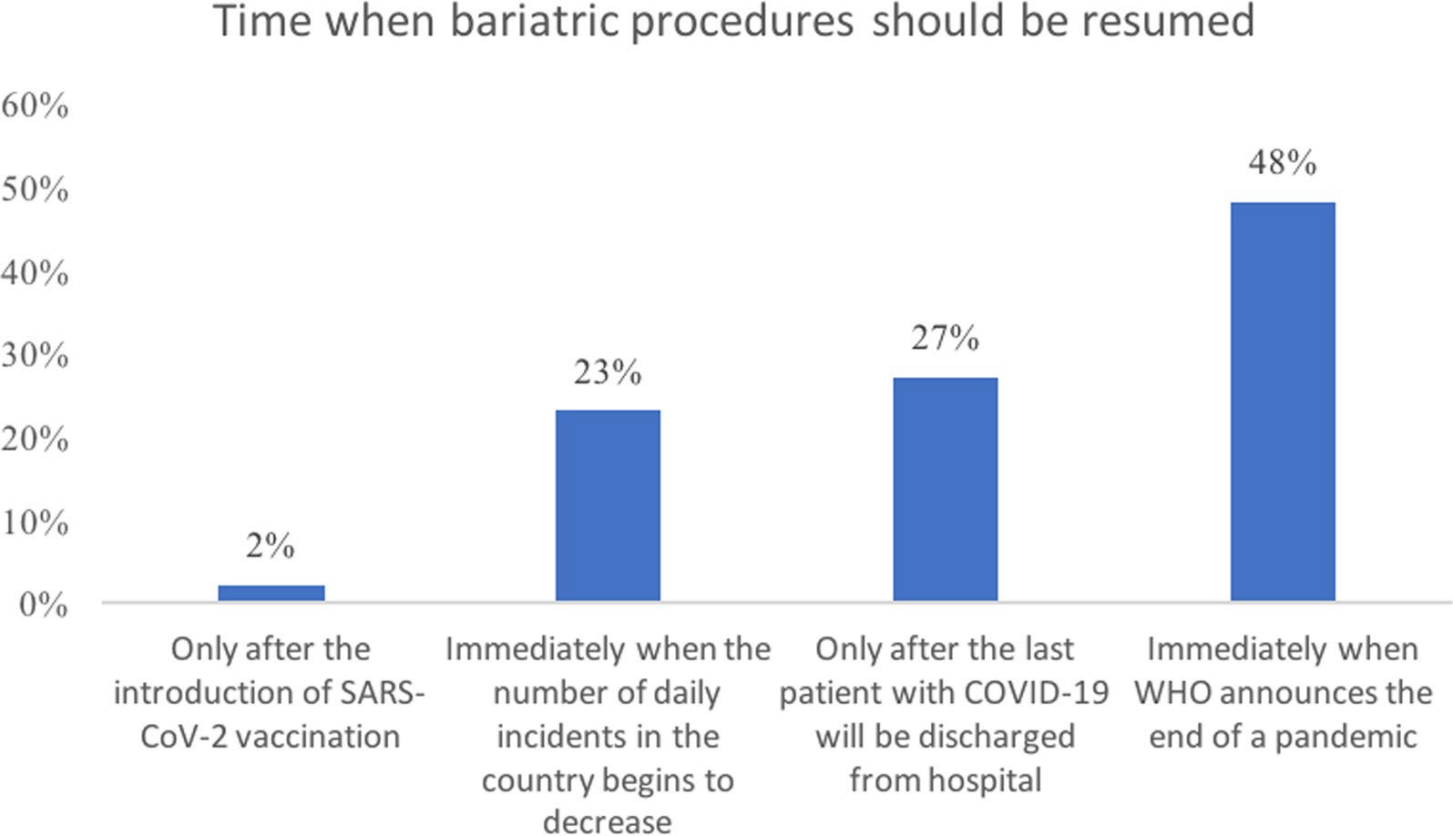
Time when bariatric procedures should be resumed

**Fig. 3:**
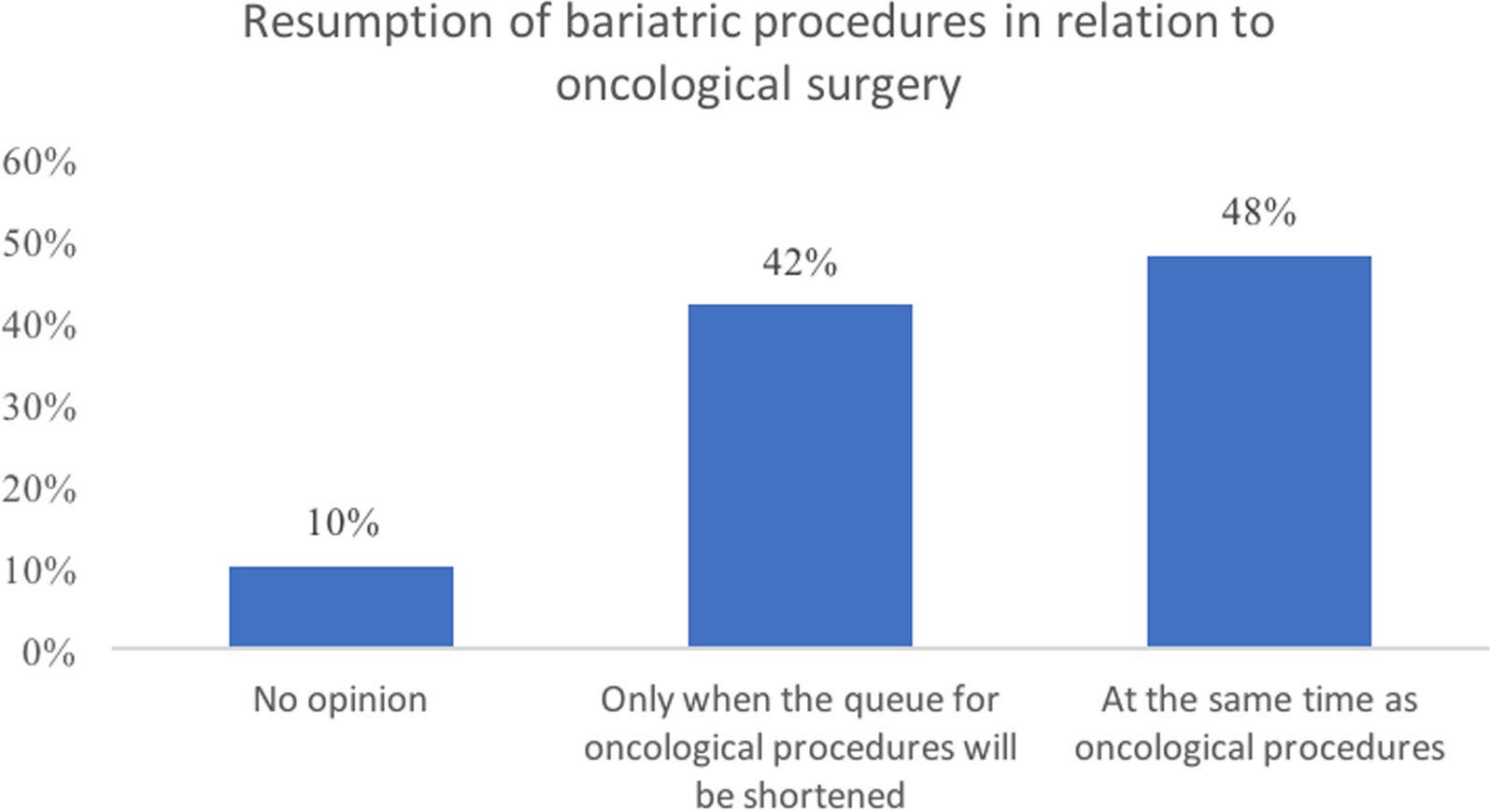
Resumption of bariatric procedures in relation to oncological surgery

In the opinion of 40 (81%) respondents, the current situation will affect future work after a pandemic and prolong waiting list for bariatric procedures. Only 9 (19%) participants declared prepared plan for post-pandemic bariatric care, 22 (45%) are currently working on it, 18 (36%) do not resolve this issue yet. Surgeons are worried about the financial aspect of bariatric procedures after pandemic—46 (94%), 3 (6%) believe that a pandemic will not affect bariatrics funding. All participants declared a high willingness to resume bariatric surgery after the SARS-CoV-2 pandemic, median score 10 (10–10). Asked when bariatric procedures should resume, respondents answered: immediately when WHO announces the end of a pandemic—24 (48%), after the last patient with COVID-19 was discharged from hospital—13 (27%), immediately when the number of daily new infections in the country starts to decrease—11 (23%), after the introduction of SARS-CoV-2 vaccination—1 (2%) (Fig. 2). In the context of oncological treatment bariatric surgery should resume in parallel—24 (48%), after managing oncological queue—20 (42%), 5 (10%) participants had no opinion (Fig. 3). 34 (69%) surgeons believed that pandemic will not affect qualification rules or types of surgery proposed to the patients in the future. The majority of participants, 44 (90%), believe that the pandemic will not affect the safety of bariatric procedures. In opinion of 10% sleeve gastrectomy will be the safest procedure after the pandemic.

## Discussion

The presented study is a result of a national survey that was dedicated for bariatric surgeons. Survey shows COVID negative impact on bariatric care in Poland. As a result of the pandemic, most bariatric procedures were postponed which is officially recommended [5, 7]. Data from around the world indicate this affects all types of surgery, including oncology [8]. This pandemic definitely represents significant harm for bariatric patients. Difficulties also arise in the functioning of the bariatric outpatient clinic. Telemedicine techniques have undoubtedly become preferred. Digital healthcare provides a necessary solution in this time of unprecedented medical crisis [9]. Our survey shows that telemedicine is possible and well suited to provide bariatric care during the pandemic.

Survey also shows how, in surgeons' opinion, SARS-CoV-2 will affect bariatric care when pandemic ends. The study showed that bariatric surgeons are ready to resume bariatric surgery as soon as possible after the pandemic. They also consider patients with different diseases who will be negatively affected during the pandemic and after it. Most healthcare systems are prioritizing surgical care for oncological patients, emergency surgery and other elective surgeries. When we combine it with the expected problems with public finances, possibly decreased reimbursement, there is a concern that bariatric patients’ treatment may have to be postponed even further [10]. Survey respondents consider that the COVID-19 pandemic will not affect the safety of future bariatric procedures.

### Limitations

This study is associated with several limitations. Multiple accesses to the questionnaire by respondents were potentially possible. To avoid the bias Authors have conducted a log-file analysis aimed to identify potential multiple entries from the same individual. Unfortunately, there still was a possibility of multiple entries from a single respondent. However, we have additionally included an instruction at the beginning of the survey and there was no incentive for sending multiple responses. The overall number of respondents is relatively low. It is important to notice, that sample size was limited only to surgeons, that are members of the Bariatric Chapter at the Association of Polish Surgeons, in which response rate has reached 60%. This constitutes a high score when compared with available literature including online-survey based studies [11–14]. Therefore, low number of respondents resulted from a limited target group of potential subjects. Moreover, we concentrated only on Polish bariatric departments, so it may be difficult to extrapolate our results to other countries, however, we expect that most bariatric surgeons have similar problems.

## Conclusions

The SARS-CoV-2 pandemic resulted in canceling or postponing of bariatric procedures in Poland. Access to bariatric care during the pandemic is limited and redirected to telemedicine. Surgeons are ready to resume bariatric operations immediately after the pandemic, but its end is difficult to determine. In the surgeons’ opinion, the pandemic will not affect the safety of bariatric surgery in the future. The extended waiting list and financial aspects will be the main issues after the pandemic. Guidelines about post-pandemic bariatric care organization plans are yet to be established.

## Supplementary Information

**Additional file 1:** Survey.

## Data Availability

The datasets used and/or analyzed during the current study are available from the corresponding author on reasonable request.

## Abbreviations

SARS-CoV-2: Severe Acute Respiratory Syndrome Coronavirus 2
WHO: World Health Organization
COVID-19: Coronavirus disease
IFSO: International Federation for the Surgery of Obesity and Metabolic Disorders
SCMiB: Metabolic and Bariatric Chapter at the Association of Polish Surgeons
