# Supplementary material for: Impact of SARS-CoV-2 pandemic on bariatric care in Poland: results of national survey
Source: BMC Surg. 2020 Dec 3;20:314. doi: 10.1186/s12893-020-00990-7 (PMC7711258; doi:10.1186/s12893-020-00990-7)
Supplement: Supplementary file 1 — Additional file 1: Survey. [file 12893_2020_990_MOESM1_ESM.docx]

**Survey**

1. Age: [fill in years]
2. Sex: Male/Female
3. Stage of surgical training: Resident/Specialist/Certificated bariatric surgeon,
4. Type of hospital – Academic/State hospital/Municipal hospital
5. Current status of the hospital during pandemic: COVID-19 dedicated hospital/ COVID-19 non-dedicated hospital
6. COVID-19 patients were treated in the hospital: yes/no
7. How many bariatric procedures were performed at your facility in 2019 (January and February): [fill in estimated number]
8. How many bariatric procedures were performed in 2020 until the pandemic was announced by WHO (January, February): [fill in estimated number]
9. Does your center currently provide care for bariatric patients in a normal extent: yes/no
10. Is your center currently performing bariatric surgery: yes/no
11. Since when exactly have bariatric operations been reduced or discontinued in your facility: [fill in date]
12. How many bariatric operations have been canceled up to date: [fill in number]
13. The discontinuation of performing bariatric procedures was dictated by: Bariatric procedures were not cancelled/Cancellation of most planned surgeries, including bariatric procedures by the hospital management/ Internal arrangements in the surgical ward/ The knowledge that obesity is an independent, additional risk factor for severe COVID-19 infection/ Canceling the surgery by the patients themselves
14. The limitation or total suspension of bariatric procedures is: [assess on a scale 1 (not necessary) to 10 (absolutely necessary)]
15. What is the current model of care for bariatric patients treated in your facility: there is no difference - the care for bariatric patients is provided as usual/ there is no possibility of providing care for bariatric patients/remote consultations via phone, skype etc./social media (chats, online lectures)/providing care with the help of patient organizations or support groups
16. How were bariatric patients qualified for surgery at your center informed about postponing the surgery: up to date there was no need for postponing the surgery/via phone/group message on social media/via website of our center/patients contacted their center independently/Patients were not informed
17. Do you consider it necessary to continue to provide remote care for bariatric patients: yes/no/i do not have an opinion
18. Please specify to what extent are bariatric patients currently being managed: bariatric patients are being consulted as usual/no consultations for bariatric patients/consultations for patients shortly after bariatric surgery (up to 3 months after the procedure)/consultations for bariatric patients including long-term follow-up/qualifying new patients for bariatric surgery/ consultations for bariatric patients aimed to prepare for the procedure
19. Please specify readiness to provide remote care for bariatric patients: [assess on a scale 1 (no preparation) to 10 (fully prepared)]
20. Are tele-consultations have been introduced for bariatric patients in your center: yes, to the full extent/yes, to a limited extent/no
21. Do you consider it necessary to continue to provide remote care for bariatric patients: [assess on a scale 1 (not necessary) to 10 (absolutely necessary)]
22. In the current situation, I consider remote consultations provided by a surgeon as: [assess on a scale 1 (not necessary) to 10 (absolutely necessary)]
23. In the current situation, I consider remote consultations provided by a dietetician as: [assess on a scale 1 (not necessary) to 10 (absolutely necessary)]
24. In the current situation, I consider remote consultations provided by a psychologist as: [assess on a scale 1 (not necessary) to 10 (absolutely necessary)]
25. I consider the participation of support groups and patient organizations during a pandemic to be: [assess on a scale 1 (not necessary) to 10 (absolutely necessary)]
26. Are "remote consultations" during a pandemic fully accepted by patients: [assess on a scale 1 (not accepted) to 10 (fully accepted)]
27. Are patients satisfied with this form of medical advice: [assess on a scale 1 (not satisfied) to 10 (completely satisfied)]
28. Are your patients currently performing all the tests you have ordered: yes/no/i do not know
29. In the current situation, does your center provide necessary assistance in the case of emergency in patients after bariatric surgery: yes, to the full extent/yes, to a limited extent (referring patients to other centers)/no
30. Do your patients express concern about their health in the context of the COVID-19 pandemic: yes/no/i do not know
31. Do your patients express the need for more support: yes/no/i do not know
32. Do your patients express the need for better access to information on the threat to their health and life in the context of the COVID-19 pandemic: yes/no/i do not know
33. In the group of bariatric patients, do older people report more concerns about their health during a pandemic: yes/no/i do not know
34. Do your patients perform any form of physical activity during periods of limited outside physical activity due to the need to stay at home: yes/no/i do not know
35. During contact with patients, do you suggest them changes in their daily diet, e.g. increase in protein supply, etc.: yes/no/i do not know
36. Do you inform patients suffering from obesity that their disease is an important risk factor worsening the course of COVID-19 infection: yes/no/i do not know
37. How will the current situation affect your ward's activity as a bariatric facility: no significant impact/ it will influence the functioning of the center, but it will not elongate the waiting period for bariatric surgery/it will influence the functioning of the center and it will elongate the waiting period for bariatric surgery/bariatric procedures will be suspended until waiting periods for other surgical procedures will l normalize
38. Has your center manage to create a plan for a large number of elective procedures that have been postponed: yes/no/we are currently working on it
39. Please indicate your readiness to resume bariatric surgery following a pandemic: [assess on a scale 1 (not ready) to 10 (fully ready)]
40. Bariatric procedures should be resumed: immediately when the number of daily new infections in the country begins to decrease/only after the last patient with COVID-19 has been discharged from hospital/immediately when WHO announces the end of a pandemic/only after the introduction of SARS-CoV-2 vaccination
41. Bariatric procedures should be resumed: due to the low risk of complications, they should be resumed before oncological procedures/at the same time as oncological procedures/only after some time, when the waiting period for oncological procedures will be shortened/i do not have an opinion
42. Will SARS-CoV-2 pandemic affect future qualification guidelines for bariatric surgery: yes/no/ i do not have an opinion
43. Will SARS-CoV-2 pandemic affect the type of bariatric surgery performed: yes/no/ i do not have an opinion
44. After the pandemic, the safest bariatric procedure in your opinion will be: SG/RYGB/OAGB/AGB/I believe that a pandemic will not affect the safety of bariatric procedures
